# Supplementary figures and images for: Deep proteomic analysis of obstetric antiphospholipid syndrome by DIA-MS of extracellular vesicle enriched fractions
Source: Commun Biol. 2024 Jan 15;7:99. doi: 10.1038/s42003-024-05789-3 (PMC10789860; doi:10.1038/s42003-024-05789-3)

Supplementary Figure 1. Uncropped and unedited blot images.

Figure 1b

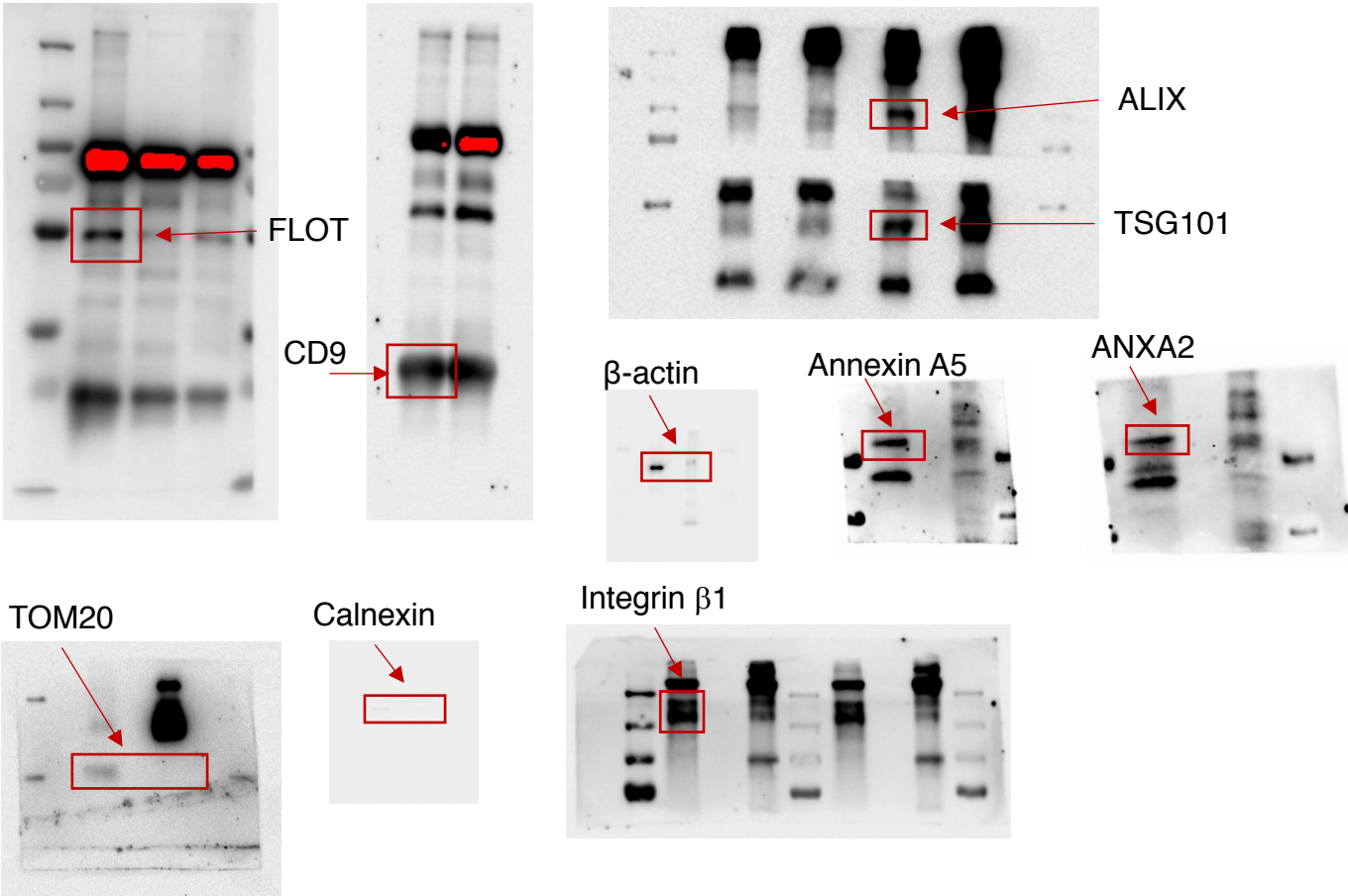

Figure 5c

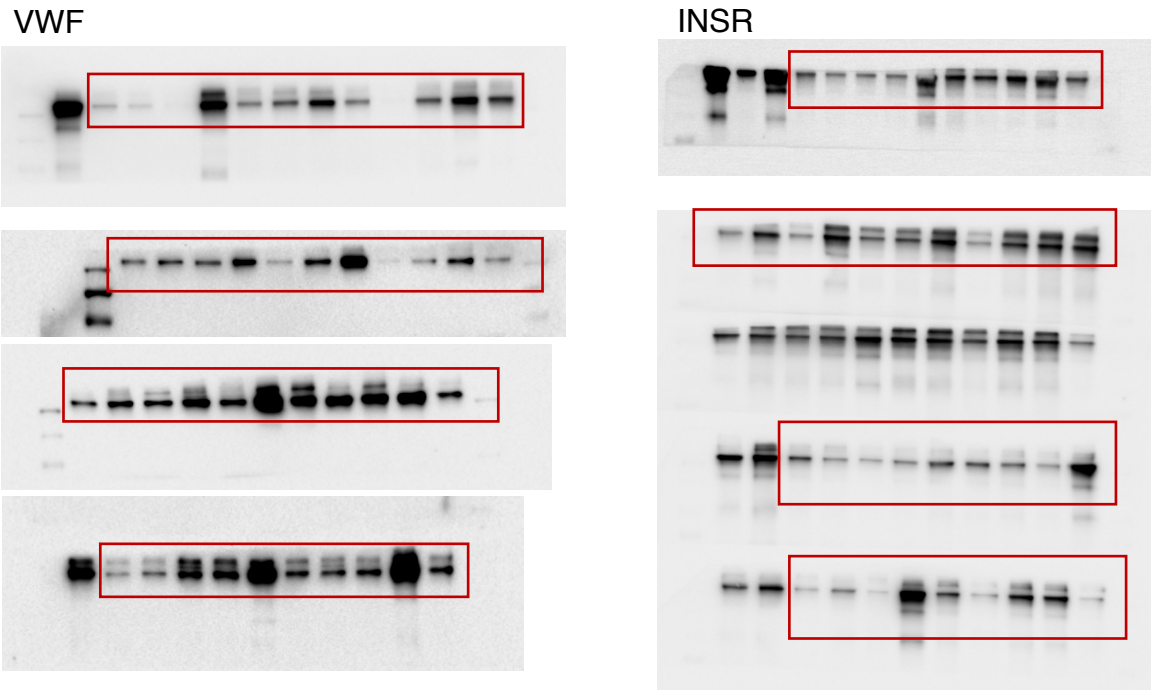

Supplement: Supplementary file 2 — Supplementary Figure 1 [file 42003_2024_5789_MOESM2_ESM.pdf]
