# Supplementary material for: Deep proteomic analysis of obstetric antiphospholipid syndrome by DIA-MS of extracellular vesicle enriched fractions
Source: Commun Biol. 2024 Jan 15;7:99. doi: 10.1038/s42003-024-05789-3 (PMC10789860; doi:10.1038/s42003-024-05789-3)
Supplement: Supplementary file 3 — Description of Additional Supplementary Files [file 42003_2024_5789_MOESM3_ESM.pdf]

Description of Additional Supplementary Files

File name: Supplementary Data 1
Description: Identified proteins and relative intensity of HPLEV from serum samples.

File name: Supplementary Data 2
Description: Identified proteins and relative intensity of HPSEV from serum samples.

File name: Supplementary Data 3
Description: Identified proteins and relative intensity of WCLEV from serum samples.

File name: Supplementary Data 4
Description: Identified proteins and relative intensity of WCSEV from serum samples.

File name: Supplementary Data 5
Description: Identified proteins and relative intensity of immunodepleted serum samples.

File name: Supplementary Data 6.
Description: Demographic information of the healthy controls and patients enrolled in Figure 5b.

File name: Supplementary Data 7
Description: Identified proteins and relative intensity of WCLEV from 44 serum samples in OAPS disease cohort.

File name: Supplementary Data 8
Description: Identified proteins and relative intensity of WCSEV from 38 serum samples in OAPS disease cohort.

File name: Supplementary Data 9
Description: Demographic information of the healthy controls and patients enrolled in Figure 5 c, d, e.

File name: Supplementary Data 10
Description: Demographic information of the healthy controls and patients enrolled in Figure 4 and Figure 5a (Samples with yellow highlight were not included in WCSEV due to failure in sample preparation).

File name: Supplementary Data 11
Description: Number of proteins identified in WCLEVs and WCSEVs Related to Figure 4b.

File name: Supplementary Data 12
Description: Coefficient of variation (CV) of the proteomic data was calculated by the proteins quantified in three quality control (QC) samples. Related to Figure 4c.

File name: Supplementary Data 13
Description: Quantitative analysis of the data in Figure 5c. Related to Figure 5d, e.
